# Luminescent gold nanoclusters for bioimaging applications

**DOI:** 10.3762/bjnano.11.42

**Published:** 2020-03-30

**Authors:** Nonappa

**Affiliations:** 1 Department of Applied Physics, Aalto University School of Science, Puumiehenkuja 2, FI-02150, Espoo, Finland,; 2 Bioproducts and Biosystems, Aalto University School of Chemical Engineering, Kemistintie 1, FI-02150, Espoo, Finland

**Keywords:** bioimaging, biosensing, gold nanoclusters, immunoassay, luminescence, self-assembly, theranostics

## Abstract

Luminescent nanomaterials have emerged as attractive candidates for sensing, catalysis and bioimaging applications in recent years. For practical use in bioimaging, nanomaterials with high photoluminescence, quantum yield, photostability and large Stokes shifts are needed. While offering high photoluminescence and quantum yield, semiconductor quantum dots suffer from toxicity and are susceptible to oxidation. In this context, atomically precise gold nanoclusters protected by thiol monolayers have emerged as a new class of luminescent nanomaterials. Low toxicity, bioavailability, photostability as well as tunable size, composition, and optoelectronic properties make them suitable for bioimaging and biosensing applications. In this review, an overview of the sensing of pathogens, and of in vitro and in vivo bioimaging using luminescent gold nanoclusters along with the limitations with selected examples are discussed.

## Introduction

Imaging methods play a central role in understanding the structural and functional biological processes of biomolecules, cells, tissues, organs, and even entire living organisms [1–2]. The importance of bioimaging in preclinical, clinical evaluation and patient treatment has encouraged extensive investigation to develop new imaging methods [3–4]. Among several imaging techniques, fluorescence microscopy has evolved as a widely used non-invasive method to visualize real-time biological processes with high spatial resolution [5–6]. The image quality of biological structures under fluorescence microscopy also depends on the performance of the fluorophores. Furthermore, bioimaging of cells and tissues faces additional challenges due to background autofluorescence generated from the intrinsic emission of biomolecules [7]. Antibodies conjugated to low molecular weight fluorescent dyes have been used for various bioimaging applications [8]. Despite their cost-effectiveness, and water solubility, organic dyes display a small Stokes shift, low photochemical stability and they undergo photobleaching [9–10]. Luminescence can also be achieved via intramolecular energy transfer between an organic ligand and lanthanide metal ions through chelation [11]. Large Stokes shift, high quantum yield and long fluorescence lifetime make lanthanide complexes excellent candidates in imaging applications [12–13]. The lanthanide complexes primarily rely on chelation of metal ions with carboxylic groups, therefore the diversity of ligand design is limited. The discovery of green fluorescent protein (GFP) led to remarkable progress in bioimaging including protein quantification, tracking, sensing as well as imaging various biochemical processes [14–17]. The large molecular mass of GFP might affect the folding process of tagged proteins, or a possible aggregation may lead to cytotoxicity. Beyond molecular and biomolecular luminescent materials, colloidal luminescent nanomaterials have gained attention in recent years [18–19]. Luminescent nanomaterials including semiconductor quantum dots, carbon dots, metal-doped nanoparticles, noble-metal nanoparticles, and organic–inorganic hybrid nanoparticles, have been studied for their ultrabright photoluminescence (PL) [20–23]. Semiconductor quantum dots (SCQDs) such as CdSe, CdTe, CdS, ZnS, ZnSe, PbS and PbSe have widely been studied as luminescent nanomaterials [24–25]. This is attributed to the possibilities to tune their size, surface functionalities, quantum confinement and high quantum yield (60–90%) [26–27]. Importantly, SCQDs display a broad spectrum of colors covering ultraviolet to near-infrared (NIR). Furthermore, SCQDs offer better sensitivity, stability against photobleaching, and a narrow spectral bandwidth compared to conventional organic dyes. However, due to their cytotoxicity, the tendency to undergo aggregation inside the cells, and easy oxidation, the extensive use in bioimaging remained a challenge. Therefore, efforts have been made to prepare silicon quantum dots (SQDs) [28]. SQDs exhibit relatively low cytotoxicity and better biocompatibility compared to SCQDs. Moreover, SQDs show broad absorption spectra, higher photostability, and the PL can be tuned from the visible to the NIR region [29]. Similar to SCQDs, SQDs undergo oxidation at room temperature and have limited water solubility.

Recently, gold nanoparticles (AuNPs) with tunable size and shape-specific physicochemical properties have emerged as attractive luminescent nanomaterials [30]. Despite the early discovery of the luminescence phenomenon in bulk gold and gold films, the PL of gold remained unexplored for several decades [31–32]. This is attributed to the low quantum yield (10^−10^), which limited the practical application and the understanding of the luminescence phenomenon in detail. The realization by Wilcoxon et al. that AuNPs with sizes of less than 5 nm show luminescence has provided the foundation for extensive investigation on various nanostructures [33]. Since the 1990s, several groups have reported AuNPs, including gold nanorods showing luminescence that is million-fold higher than that of the bulk metal [34–37].

**Figure 1:**
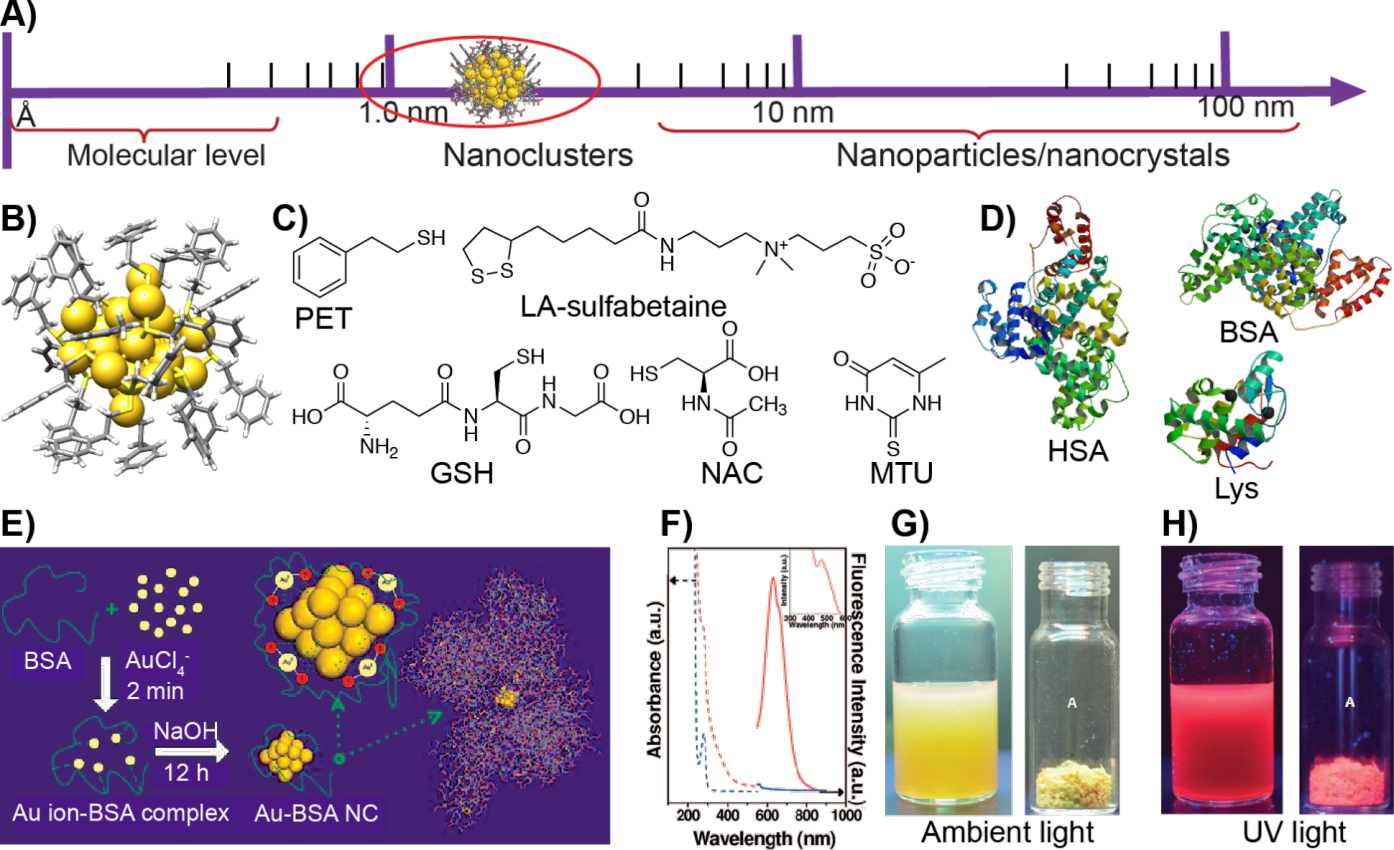
Structure, synthesis and properties of gold nanoclusters. A) Nanoclusters provide a link between molecular complexes and plasmonic nanoparticles. B) X-ray structure of [Au_25_(PET)_18_], from CSD entry JACVOB. C) Chemical structure of some of the organic thiols discussed in this review (PET: phenylethane thiol; LA: lipoic acid; GSH: glutathione; NAC: *N*-acetylcysteine; MTU: 5-methyl-2-thiouracil). D) Structures of biomolecular ligands (HSA: human serum albumin, PDB ID IE78; BSA: bovine serum albumin, PDB ID 3VO3*,* and Lys: lysozyme, PDB ID 3WUN). E) Schematic representation showing Au-BSA NC synthesis. F) Absorbance and fluorescence spectra of Au-BSA NCs. G, H) Photographs of Au-BSA NCs in solution and in the solid state under ambient light and UV light, respectively. Figure panel 1E is adapted and panel 1F is reused with permission from [63], copyright 2009 American Chemical Society. Figure panels 1G,H are adapted with permission from [64], copyright 2016 American Chemical Society.

The ability to control the properties by tuning the particle shape and surface functionalities further advanced the applications of AuNPs in a wide range of research fields. However, fundamental challenges remained related to their aggregation tendencies, polydispersity and difficulties in controlling the directionalities. In this context, atomically precise gold nanoclusters (AuNCs) opened a new opportunity for the field of colloidal science [38–40]. Atomically precise NCs with a core diameter below 3 nm contain an exact number of metal atoms and surface ligands (Figure 1A,B). Therefore, NCs are considered as colloidal molecules. Similar to plasmonic nanoparticles, the stability of NCs can be controlled by ligand passivation using small molecules, synthetic polymers or biomacromolecules. A significant difference between plasmonic AuNPs and AuNCs can be stated regarding the sensitivity. For observable changes to occur in physicochemical properties of plasmonic NPs, at least a layer of atoms needs to be removed (ca. 0.5 nm), whereas NCs already display remarkable changes after addition or removal of a single atom. Additionally, due to covalently bound ligands, the NCs show extraordinary stability under ambient conditions. While plasmonic AuNPs display size-dependent surface plasmon resonance (SPR), NCs display characteristic molecule-like electronic spectra. This is attributed to the small size and quantum confinement, and the evolution of continuous or quasicontinuous bands (of bulk gold) into discrete electronic states [40]. Another attractive property of AuNCs is photoluminescence (PL), a phenomenon that is much less understood than the surface plasmon resonance of plasmonic gold nanoparticles.

## Review

### Luminescent gold nanoclusters

Luminescent AuNCs show high photostability and biocompatibility and are nontoxic [41]. Their size is highly precise and small compared to QDs, offering a better internalization in cells and tissues [42–47]. The presence of surface ligands allows for a selective modification and biomolecular tagging. Therefore, AuNCs find potential applications in sensing, photodynamic therapy, labeling and bioimaging. However, there are challenges because the number of luminescent gold NCs is limited and the PL quantum yield is low compared to organic dyes, lanthanide complexes and SCQD-based nanomaterials. To improve the quantum yield and PL, various approaches have been developed including ligand engineering, selective doping to create alloy clusters, aggregation-induced emission, selective etching and self-assembly [48–58].

Ligands play an important role in the luminesce properties of NCs [48,55]. For example, a phenylethane thiol (PET)-capped organically soluble Au_25_ nanocluster (Au_25_PET_18_) showed similar behavior as Au_25_NCs capped with long-chain alkanes [59]. However, it was shown that by choosing the appropriate surface ligands and introducing a proper steric nature, the metal-to-ligand charge transfer can be tuned [60]. As a consequence, the PL of Au_25_ NCs has been enhanced. The electron-donor ability of ligands and the direct donation of the delocalized electron from electron-rich atoms of the ligands to the metallic kernel affect the PL of the NCs [61]. For example, for Au_25_ NCs, the PL intensity increased with increasing the charge-donor capacity of the ligands in the order of HSC_2_H_4_Ph > HSC_12_H_25_ > HSC_6_H_13_. Furthermore, it was demonstrated that the PL was enhanced by 6.5 times for 2-naphthalen-2-yl ethanethiolate-capped Au_25_ NCs compared to that of PET-capped Au_25_ NCs [43]. Water-soluble glutathione-capped [Au_25_(SG)_18_] NCs have been shown to display higher PL than the [Au_25_(PET)_18_] counterpart. Partial ligand exchange can also lead to enhanced PL in certain AuNCs. Shibu et al. partially exchanged the glutathione ligands in Au_25_(SG)_18_ NCs with three different ligands [62]. It has been shown that in the case of 3-mercapto-2-butanol (MB)-substituted products [Au_25_(MB)*_x_*(SG)_18−_*_x_*] the PL spectra exhibit a blueshift in excitation and emission compared the initial [Au_25_(SG)_18_] nanocluster. However, no significant changes were observed in *N*-acetylglutathione (NAGSH) and *N*-formylglutathione (NFGSH) ligands. Xie and co-workers reported the synthesis of a bovine serum albumin (BSA)-protected water-soluble Au_25_ nanocluster (Au-BSA) having a red emission at ca. 640 nm with a PL quantum yield of 6% (Figure 1E,F) [63]. More importantly, the Au-BSA NCs are stable under ambient conditions and retain their PL even after drying (Figure 1G,H) allowing for long-term storage [64]. Several other proteins including lysozyme, human serum albumin (HSA) and insulin have been used to prepare inherently luminescent AuNCs [65–70]. Aggregation-induced emission (AIE) is another approach where non-emissive or weakly luminescent molecules or particles emit intensely upon aggregation, boosting quantum yields by two orders of magnitude. A majority of the chromophores show high luminescence in their dilute solutions. However, in the solid state, due to aggregation-caused quenching, they turn less emissive. Luo et al. in 2001 have shown that when water was added to a solution of 1-methyl-1,2,3,4,5-pentaphenylsilole in ethanol, it turned intensely emissive [71]. The quantum yield of silole increased by 333 times in a water/ethanol (90/10 v/v) mixture compared to that of in pure ethanol solution. In solution, the dynamic intramolecular rotation serves as a route for nonradiative relaxation process. Upon aggregation, the intramolecular rotations are restricted, which blocks the non-radiative pathways and opens the radiative decay channel resulting in highly emissive aggregates [72]. The AIE phenomenon has been observed in several organic compounds of low molecular weight and in polymers [73]. The aggregation-induced luminescence of NCs has been achieved either using solvent-induced aggregation or addition of additives such as ionic polymers, proteins or peptides [74–76]. Recently, Dichiarante et al. reported NIR-luminescent AuNCs bearing superfluorinated (SF) ligands with strong emission at 1050 nm with a quantum yield of 12% [77]. An extensive account of the PL of NCs is beyond the scope of this review and has been previously summarized in several reports [54,78–82]. This review discusses an overview of the application of gold NCs in biosensing and bioimaging. Importantly, the sensing of pathogenic bacteria and viruses, in vitro imaging of cell lines and in vivo bioimaging using animal models are presented. In each section representative early examples along with the recent examples are discussed.

### Biosensing and imaging of pathogens

**Figure 2:**
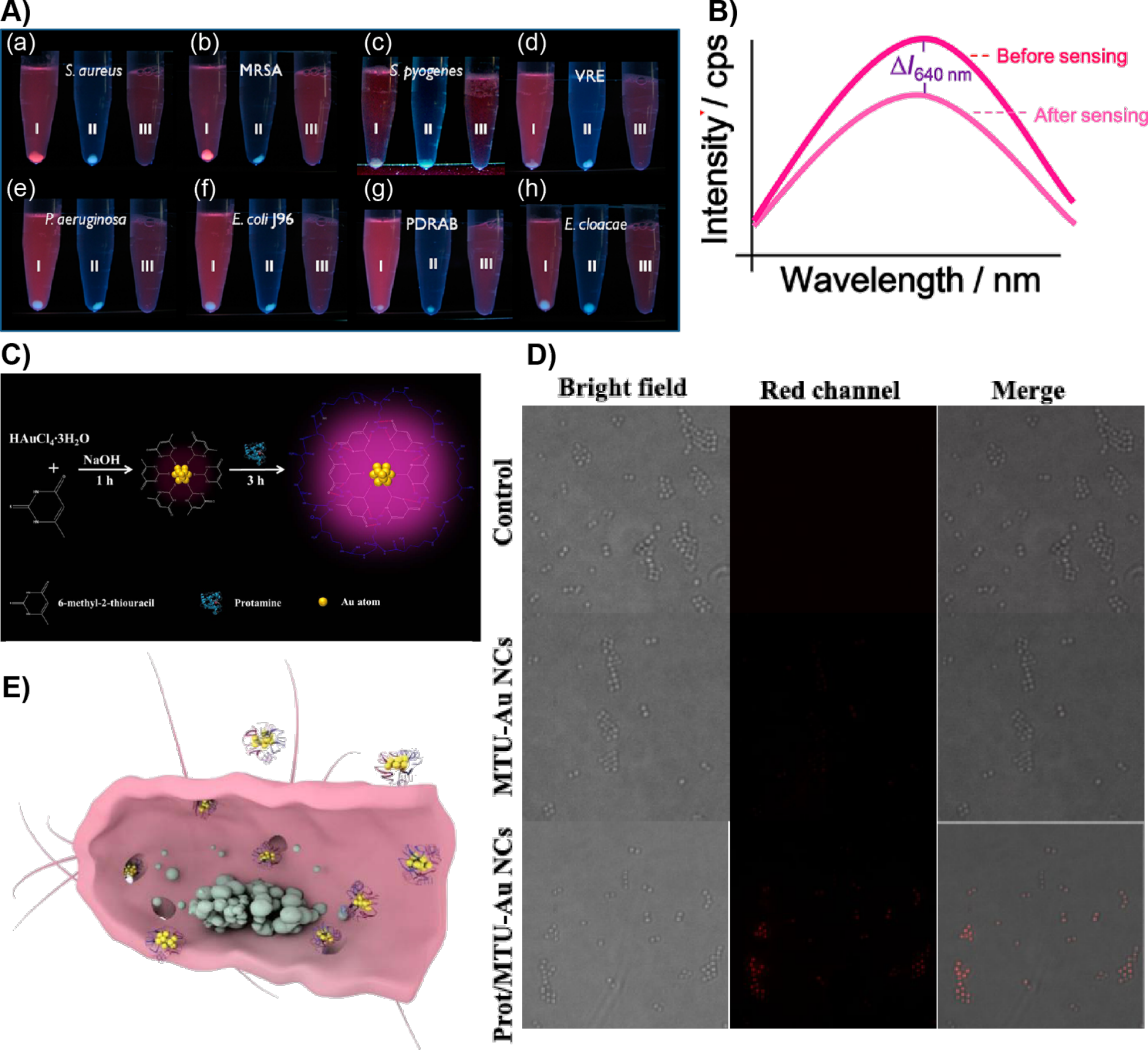
AuNC-based pathogen sensing and imaging. A) (a–h) Photographs showing the sensing of various pathogenic bacterial strains after incubating them with 0.12 mg/mL of Au-HSA NCs followed by centrifugation at 3500 rpm (tubes labeled as “I”) in PBS buffer at pH 6.0. Tubes labeled as “II” in each panel contained only bacteria. Tubes labeled as “III” in each panel show the solution containing only Au-HSA NCs. B) Fluorescence spectra showing a change in the fluorescence intensity at 640 nm of Au-HSA NCs after bacterial sensing. C) Schematic representation of Au-MTU/Prot NC synthesis. D) Microscopy images of *S. aureus* after treatment with Au-MTU/Prot NCs, Au-MTU/Prot NCs, and control. The red channel was excited at 405 nm. The images were 40 μm × 40 μm. E) A cross-sectional schematic view of a bacterium treated with Au-MTU/Prot. Figure panel 2A is adapted and panel 2B is reused with permission from [83], copyright 2012 American Chemical Society. Figure panels 2C–E are reused with permission from [84], copyright 2019 Americal Chemical Society.

Chan et al. reported human serum albumin (HSA)-stabilized gold NCs (Au-HSA) for sensing *Staphylococcus aureus* (SA) and methicillin-resistant *Staphylococcus aureus* (MRSA) bacterial strains [83]. The resulting Au-HSA NCs showed reddish PL. The ability of HSA to bind and chelate various ions as well as small molecules was exploited to design a NC-based assay. The directly synthesized Au-HAS NCs showed binding affinities for SA and MRSA strains (Figure 2). A systematic study used several other pathogenic bacteria, including *Streptococcus pyogenes*, vancomycin-resistant *Enterococcus faecalis* (VRE), *E. Coli* J96, *Pseudomonas aeruginosa*, pandrug-resistant *Acinetobacter baumannii* and *Enterobacter cloacae* in phosphate-buffered saline (PBS) at pH 6 (Figure 2A). Importantly, a reddish precipitate was formed when the Au-HSA NCs interacted with SA and MRSA. Whereas for all other pathogens a pale blue precipitate was obtained at the bottom of the Eppendorf tubes after slow centrifugation. The detection limit using Au-HSA NCs was 4.2 × 10^8^ cells/mL, which is two orders of magnitude lower than that without sensing probes in the bacterial samples. Further, Au-HSA NCs can interact with a target analyte in complex biological samples as demonstrated using urine samples containing SA strains. However, this sensing approach was not able to distinguish SA and MRSA. Therefore, sensing combined with MALDI–MS was utilized to distinguish SA and MRSA based on MALDI–MS fingerprinting. Finally, using the principal component analysis (PCA) method two different strains were qualitatively distinguished.

The possible explanation of a selective binding purely based on electrostatic interaction was ruled out. Instead, the authors assumed that it might be a specific peptide motif of HSA that interacts with the bacterial cell wall. The trypsin digestion of Au-HSA NCs was studied and various fragments were identified using MALDI–MS. To confirm further whether the peptides can interact with *S. aureus*, DVFLGRGGGC (Pep10) and RHPDYSVVLLLRGGGC (Pep16), containing the sequences no. 348 to 352 and no. 361 to 372, were synthesized and used for the synthesis of Au-Pep10 and Au-pep16 NCs. Interestingly, both Au-pep10 and Au-pep16 NCs yielded similar results suggesting that these peptides are responsible for binding. However, there was no significant binding with only one of the peptides or only HSA. Similarly, sufficient binding was not observed when a control experiment was performed with Au-BSA NCs. The above experiments suggest that in Au-HSA NCs, HSA might adopt a conformation that assists better binding.

Zhu et al. reported a rigid host–guest assembly to improve the PL of AuNCs, their antibacterial activity and bioimaging [84]. In their work, 5-methyl-2-thiouracil (MTU)-capped AuNCs (Au-MTU) were prepared. The Au-MTU NCs were then treated with protamine (Prot), a cationic peptide capable of penetrating bacterial biofilms with abundant arginine residue. The hydrogen bonding between the MTU ligands on the surface of Au-MTU NCs and the arginine residues in protamine form a supramolecular host–guest complex, i.e., Au-MTU/Prot. The supramolecular host–guest interactions rigidify the surface resulting in a 28-fold increase in the PL of Au-MTU/Prot NCs compared to that of Au-MTU NCs. The resulting Au-MTU/Prot NCs displayed antibacterial properties with abilities to kill both Gram-positive and Gram-negative bacteria, which was shown using *E. coli* and SA strains. The addition of protamine also lowered the minimum inhibitory concentration by two orders of magnitude. This is attributed to the enhanced catalytic activity upon binding with protamine, which resulted in altered oxidative stress and a higher generation of reactive oxygen species (ROS).

**Figure 3:**
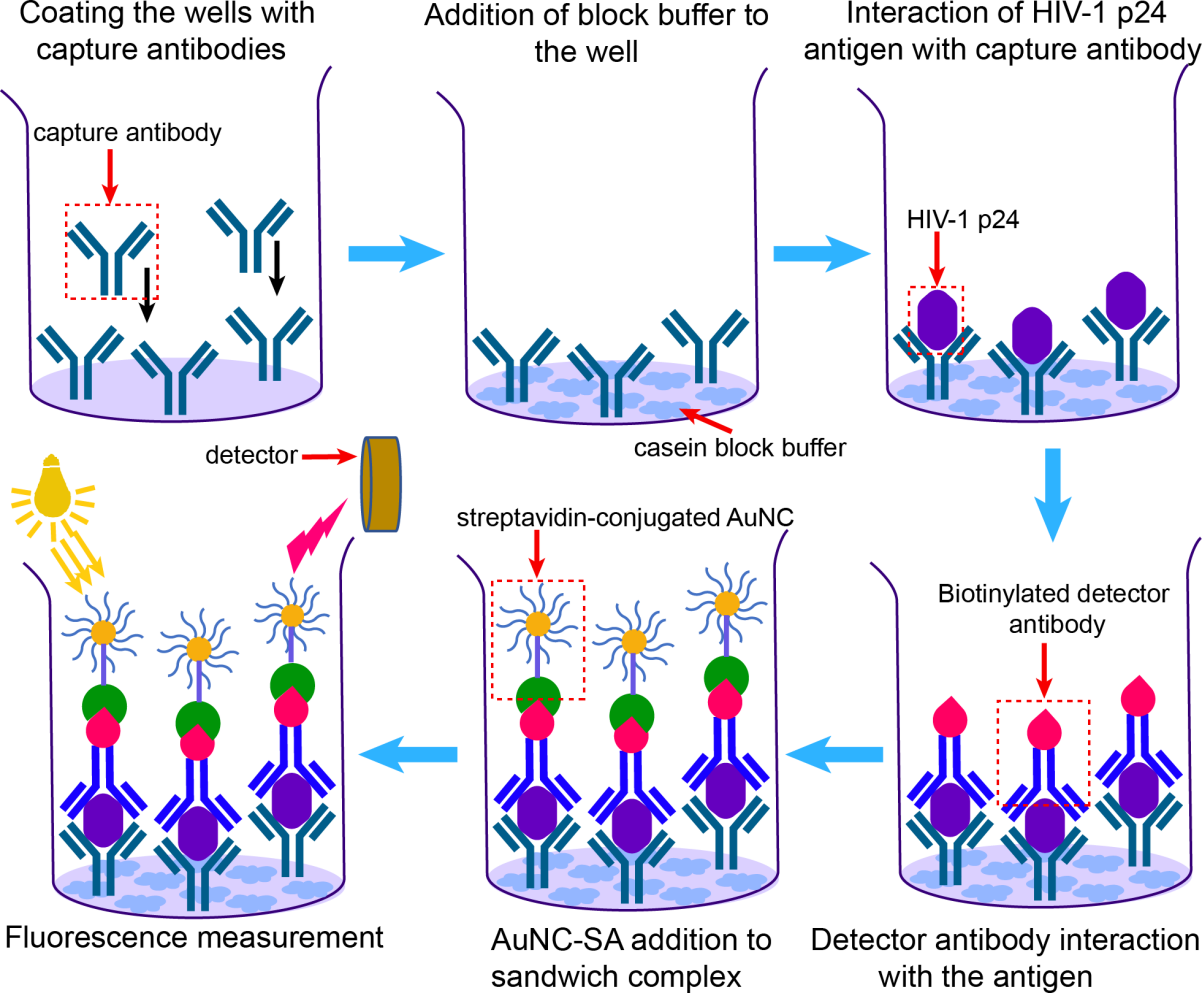
Schematic representation of AuNCIA in the detection of HIV-1 p24 antigen inspired by [85].

Kurdekar et al. developed a fluorescent gold nanocluster immunoassay (AuNCIA) for early and sensitive detection of human immune deficiency virus (HIV) infection in vitro and HIV-infected patient samples [85]. For this study, glutathione-capped AuNCs were coupled with streptavidin (Au-SA) using EDS/NHS coupling. The strong noncovalent interaction between streptavidin and biotin was exploited. To achieve the immunoassay, an antibody–antigen–antibody sandwich approach was utilized (Figure 3). The substrates were first coated with capture antibodies that will interact strongly with HIV-1 p24 antigen, a target viral protein expressed in abundance in the early stages of HIV infection. Then, a biotinylated detection antibody was added, which resulted in a sandwich complex leaving the biotin accessible for streptavidin binding. Finally, the interaction between the biotin in the detector antibody and the streptavidin in Au-SA NCs allowed for rapid detection. It was shown that AuNCIA has an analytical sensitivity at the picogram level and the sensitivity is equivalent or even better than that of other colorimetric assays such as enzyme-linked immunosorbent assay (ELISA). The AuNCIA is specific for HIV, which was shown by spiking Hepatitis C viruses with HIV-1 p24 antigen. The clinical validation using samples from HIV-positive tested patients also demonstrated the efficacy of AuNCIA detection and no false negatives were observed. This suggests that AuNCs with appropriate labeling and surface functionalization offer new avenues for rapid detection and the development of new immunoassays.

### Imaging and labeling mammalian cell lines

**Figure 4:**
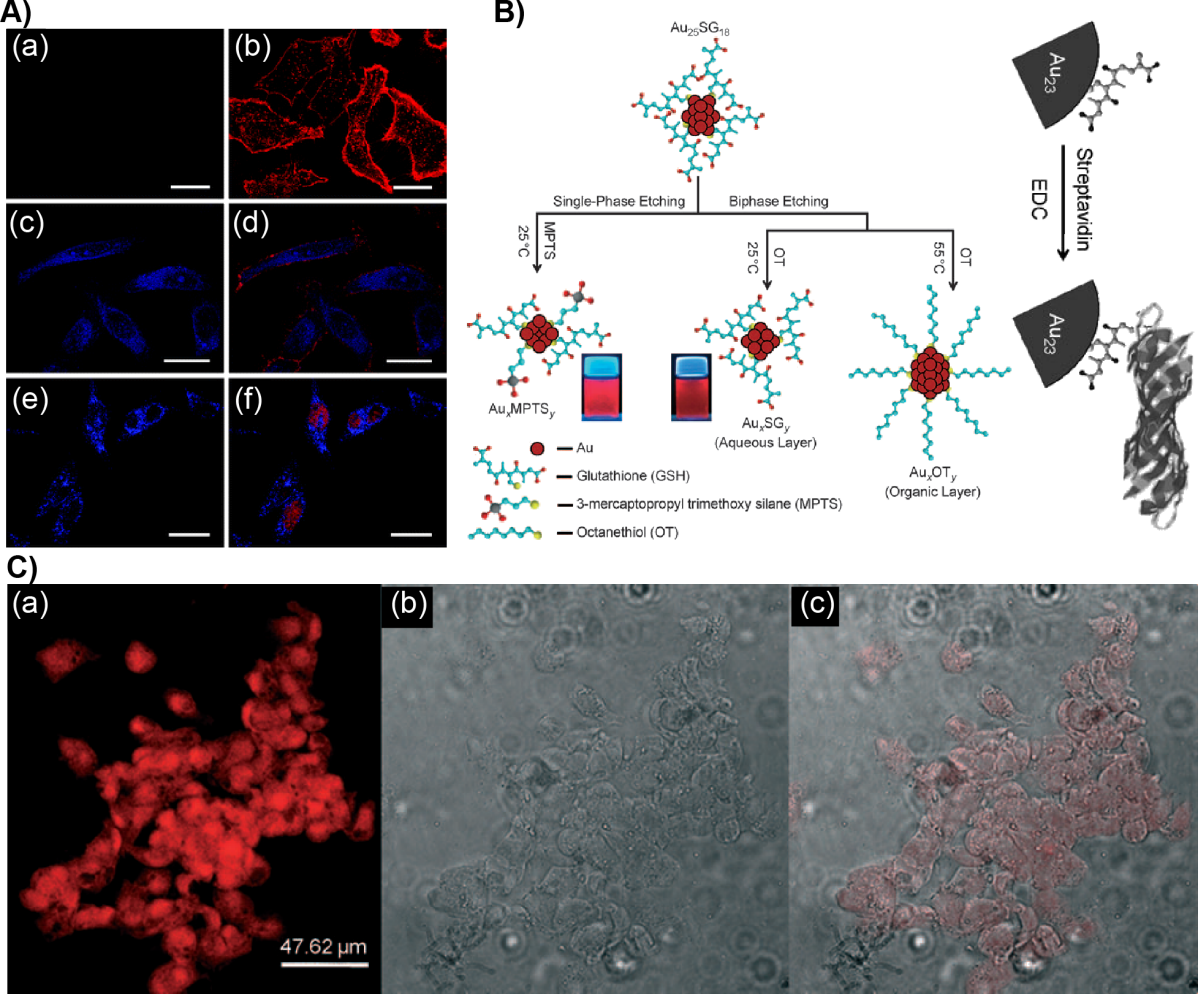
Cellular labeling and imaging using AuNCs. A) HeLa cells were treated with (a, b) Au-MUDA NCs and (e, f) Au-MUDA-NLS NCs for 1.5 h. The left panel shows the one-color image. The right panel shows the two-color colocalization image of HeLa cells incubated with Au-MUDA-NLS NCs and counterstained with membrane dye WGA–Alexa 594 and nuclear dye SYTO 59; scale bar: 25 μm. B) Schematics showing the etching method to prepare luminescent AuNCs and their conjugation with streptavidin. C) (a) Fluorescence, (b) bright-field, (c) and overlay of fluorescent and bright-field images of human hepatoma (HepG2) cells stained with streptavidin-conjugated Au_23_ NCs. Figure panel 4A is adapted with permission from [86], copyright 2008 The Royal Society of Chemistry. Figure panel 4B is reused and panel 4C is adapted with permission [87], copyright 2009 Wiley‐VCH Verlag GmbH & Co. KGaA, Weinheim.

Beyond their antibacterial effect and pathogen sensing, the surface functionalities of NCs allow for selective labeling for the detection of biomolecules, intracellular metal ion sensing, live-cell imaging as well as cellular apoptosis studies. Lin et al. reported 11-mercaptoundecanoic acid (MUDA)-capped AuNCs (Au-MUDA) as luminescent probes for nuclear targeting and intracellular imaging [86]. The Au-MUDA NCs were conjugated with SV40 (PKKKRKV), a specific peptide for nuclear-localization signal (NLS). The Au-MUDA-NLS NCs were easily internalized and distributed in the nucleus when studied using HeLa cell lines (Figure 4A). The intracellular and nuclear distribution was studied using a membrane dye WGA-Alexa 594 and a nuclear dye, SYTO59. This suggested that Au-MUDA-NLS NCs were well distributed in the cytoplasm as well as in the nucleus. Muhammed et al. reported brightly NIR-emitting Au_23_ and Au_25_ NCs using single-phase and biphasic etching of [Au_25_(SG)_18_] (Figure 4B) [87]. The Au_23_ clusters were selectively conjugated with streptavidin for a specific labeling of cells. Here the strong binding of streptavidin with biotin was exploited for imaging human hepatoma cells (HepG2, Figure 4C). HepG2 are cancerous cells that contain excess biotin.

Retnakumari et al. studied the surface functionalization of Au-BSA NCs with folic acid (FA) for selective binding, internalization and imaging of folate receptor-positive (FR^+^) oral squamous cell carcinoma (KB) and breast cancer adenocarcinoma MCF-7 cell lines [88]. Since then, there have been numerous other reports that have shown various surface modifications to image a wide variety of cell lines.

**Figure 5:**
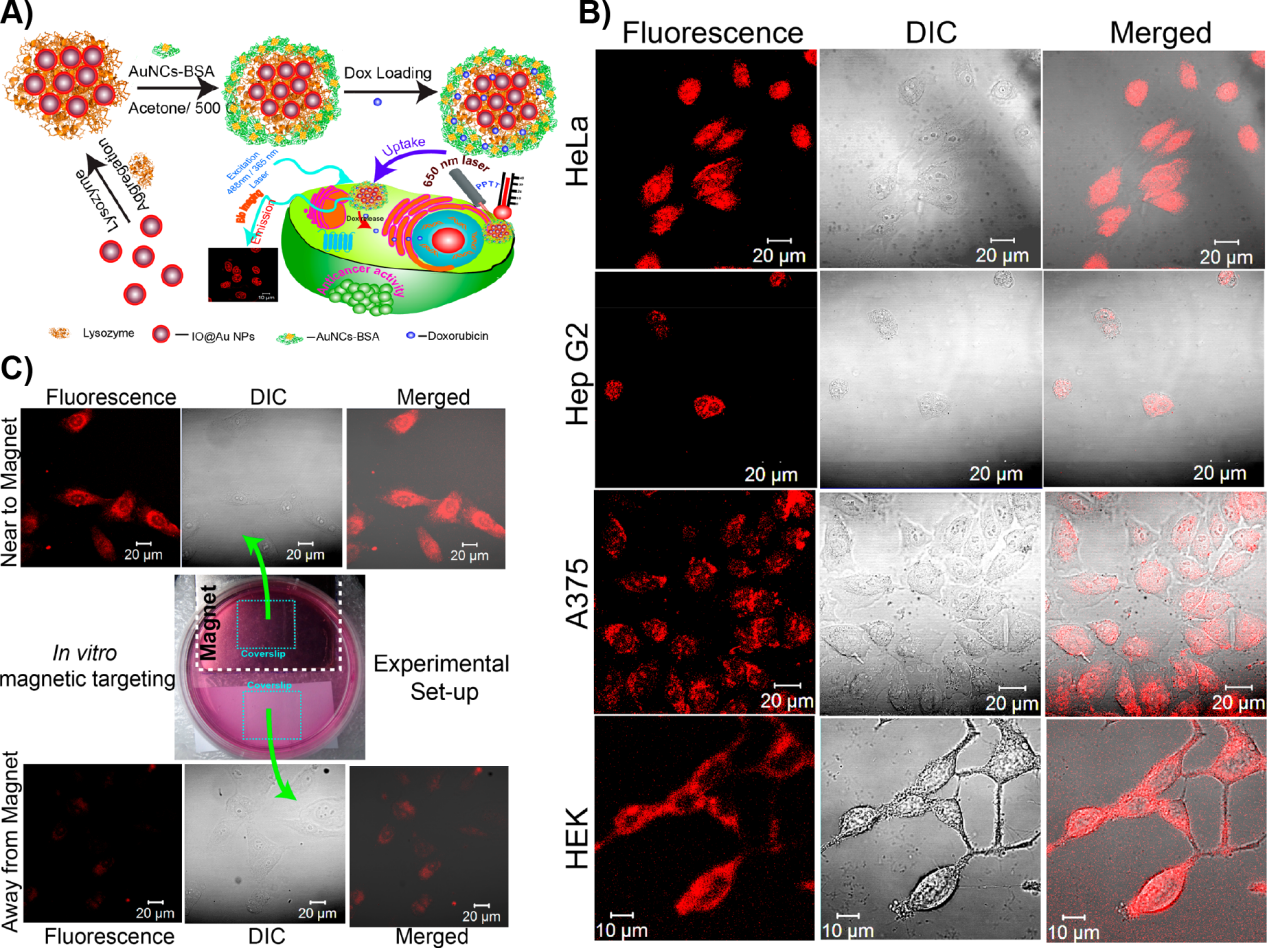
Plasmonic magnetoluminescent agglomerates. A) Schematic representation of the fabrication of the PML-MF nanocarriers and their application in photothermal therapy. B) CLSM images of HeLa, HepG2, A375, and HEK cells treated with the PML-MF nanocarrier for 2 h; images were recorded with a 488 nm excitation laser. C) In vitro magnetic targeting of HeLa cells treated with the PML-MF nanocarriers. Figure panels 5A–C are reused with permission from [90], copyright 2019 Americal Chemical Society.

Pan et al. reported composite core–shell nanoparticle–nanocluster agglomerates as luminescent nanocarriers for imaging and combination therapy [89–90]. Core–shell nanoparticles consisting of oleic acid-capped superparamagnetic iron oxide nanoparticles (IONPs, *d* = 6.7 ± 1.2 nm) were used (Figure 5A). The IONPs were subsequently coated with a gold shell using the citrate reduction of Au(III) salts resulting in core–shell (IO@Au) nanoparticles of 9.3 ± 2.6 nm. The core–shell particles underwent lysozyme-mediated aggregation (IO@Au-Lys). The aggregated structures were further treated with Au-BSA NCs (IO@Au-Lys-Au-BSA) to form a composite structure. The combination allowed for plasmonic and magnetic resonance, and luminescence in a single composite system for plasmonic photothermal therapy (PPTT). The bioimaging capability of the plasmonic magneto-luminescent multifunctional nanocarrier (PML-MF) systems were studied in vitro using three types of cancer cells, namely, HeLa, HepG2 and A375, as well as a normal HEK cell line (Figure 5B). Confocal imaging confirmed the internalization of the nanocarriers. After incubating the cell lines with sodium azide, there was a decrease by 82% of uptake of the nanocarriers, suggesting that the internalization is through endocytosis. The superparamagnetic nature of the PML-MF allowed for the magnetic targeting of the nanocarriers. Further, the ability of BSA to encapsulate drug molecules was explored to load doxorubicin (DPML-MF) in the nanocarriers.

The release kinetics of doxorubicin studied at pH 7.4 and 4.4 were found to be identical with a fast release up to 6 h and a slow release up to 20 h in PBS buffer, possibly due to diffusion-driven drug release. DPML-MF remained stable in human blood serum up to 24 h. DPML-MF showed a significant effect on HeLa, HepG2 and A375 cell lines with IC_50_ values 200-fold higher compared to that of free doxorubicin, presumably due to slow release from the nanocarriers. Alternatively, a significant killing efficiency of HeLa cells was achieved using just 0.46 μg/mL of free doxorubicin in combination with 200 μg/mL of PML-MF and laser irradiation for 10 min, further showing the potential for photothermal therapy. While PML-MF alone was not toxic to healthy HEK cell lines, the treatment with DPML-MF showed a similar antiproliferative effect on healthy cell lines as that of cancerous cells. Therefore, the selective killing of cancer cells was not achieved. The superparamagnetic property of the nanocarriers also allowed for magnetic targeting (Figure 5C). In another recent study, Pan et al. using glutathione-capped AuNCs showed that the aggregation-induced emission could be sensitive to the viscosity of the medium and that can potentially be used for intracellular viscosity imaging [91].

**Figure 6:**
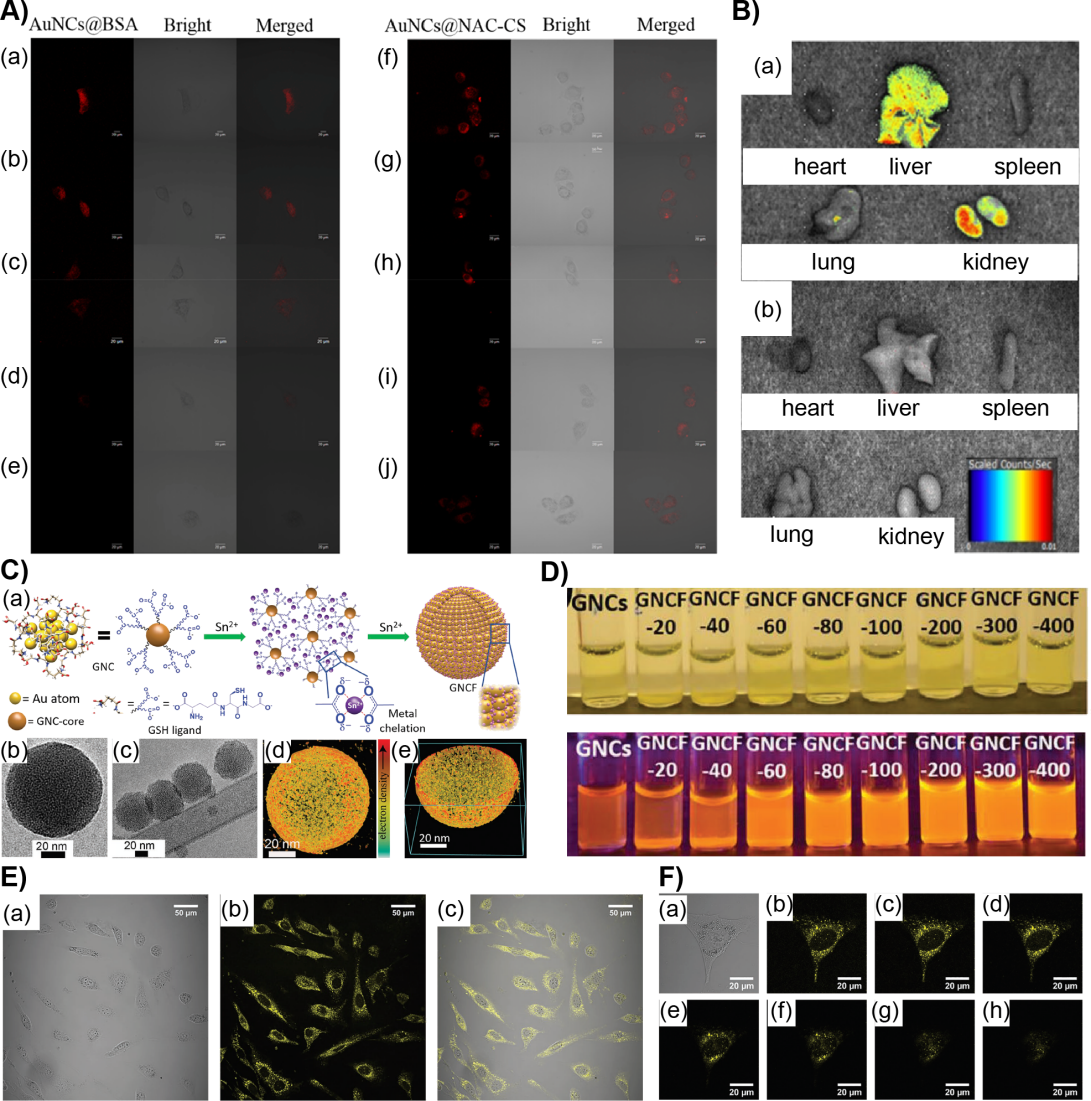
A) Confocal fluorescent microscopy images showing the metabolism of Au-BSA NCs and Au-NAC-CS NCs in living Hela cells beginning 1 h after incubation and imaged at (a, f) 0 h, (b, g) 1 h, (c, h) 2 h, (d, i) 4 h and (e, j) 8 h. B) Ex vivo fluorescence images of isolated organs (heart, liver, spleen, lung, kidney) from (a) mice 6 h after injection and (b) isolated organs from the untreated mice. C) (a) Schematics of AuNCF synthesis, (b, c) cryo-TEM images and (d, e) electron tomography of the AuNCF superstructure. D) Photographs under ambient light (top) and UV light (bottom) with varying concentrations of SnCl_2_ added to Au-GSH NCs. E) Confocal microscopy images of NIH3T3 cell lines incubated with AuNCFs for 1 day (a) bright-field image, (b) confocal fluorescence image and (c) merged image of a cell. F) Image of a single NIH3T3 cell and its *Z*-stacks (a–h) with 1.0 μm intervals of the same cell. Figure panels 6A,B are adapted with permission from [92], copyright 2018 American Chemical Society. Figure panels 6C,D are reused and panels 6E,F are adapted with permission from [53], copyright 2019 Wiley‐VCH Verlag GmbH & Co. KGaA, Weinheim.

Recently Duan et al. reported the synthesis of NIR-luminescent AuNCs capped with *N*-acetyl-ʟ-cysteine (NAC-CS) for long-time imaging [92]. The Au-NAC-CS NCs were insensitive to hydrogen peroxide and trypsin in contrast to Au NCs coated with BSA or other proteins, allowing for extended imaging times in HeLa cells (Figure 6A). When incubated with HeLa cell lines up to 4 h, strong fluorescence was observed. Unlike Au-BSA NCs, even after 8 h, weak fluorescence was still observed. Biodistribution studies of Au-NAC-CS NCs in different organs of mice, including heart, liver, spleen, lung and kidney, were analyzed (Figure 6B). A strong fluorescence signal appeared in the liver and kidney of normal mice after 6 h of NC injection. An apparent decrease in fluorescence after more than 6 h suggested the efficient clearance of NCs and there is no accumulation leading to cytotoxicity.

A proper functionalization of AuNCs also offers opportunities to develop NC-based radiosensitizers for cancer radiotherapy. Jia et al. reported atomically precise Au_8_ NCs capped with levonorgestrel [Au_8_(C_21_H_27_O_2_)_8_] NCs with yellow-green luminescence and a quantum yield of 58.7% as a radiosensitizer for enhanced cancer therapy [93]. The toxicity studies using human oesophageal squamous cancer cells (EC1) showed that when [Au_8_(C_21_H_27_O_2_)_8_] was used at a concentration near its IC_10_ value, the luminescence of the incubated samples increased from 0 to 8 h. The luminescence, however, disappeared after 24 h indicating endocytosis of NCs. The generation of ROS upon X-ray irradiation in the presence of [Au_8_(C_21_H_27_O_2_)_8_] significantly suppressed the tumorigenicity in vivo after one radiotherapy treatment in mouse models.

Because of their well-defined surface functionalities and small size, the dispersion behavior of NCs is similar to that of supramolecular complexes. Therefore, they are excellent building blocks to achieve the formation of hierarchical supracolloidal structures. However, there are substantial challenges as the interactions between the nanoclusters are close to the thermal fluctuation energy of the surrounding media [94]. Nevertheless, hydrogen bonding has been utilized to achieve two-dimensional (2D) and three-dimensional (3D) nanocluster superstructures [95–96]. The NC assemblies have been used to encapsulate poorly water-soluble fluorinated drugs through nanoconfinement [97]. Chandra et al. recently reported highly luminescent gold nanocluster frameworks (AuNCFs) using self-assembly through metal chelation [53]. Glutathione-capped AuNCs [Au_25_(SG)_18_] spontaneously self-assembled into spherical AuNCFs upon controlled addition of SnCl_2_ (Figure 6C). The size of the AuNCFs was tunable from 30 to 200 nm in diameter, and the luminescence increased dramatically upon framework formation. Interestingly, the quantum yield was increased from 2.5% for [Au_25_(SG)_18_] to 25% for AuNCFs. Cell counting kit 8 (CCK‐8) assay and trypan blue tests with NIH3T3 and A549 cells showed no significant cytotoxicity in vitro (Figure 6D–F). Interestingly, the NC frameworks led to a higher cell viability compared to [Au_25_(SG)_18_]. This is attributed to the fact that smaller nanoparticles produce reactive oxygen species and possibly aggregate in the cellular medium. The superstructures were also found to show excellent bioavailability and luminescence and were non-toxic. The AuNCFs frameworks, because of their highly luminescent nature, also allowed for better imaging compared to [Au_25_(SG)_18_]-treated cells.

### In vivo bioimaging

**Figure 7:**
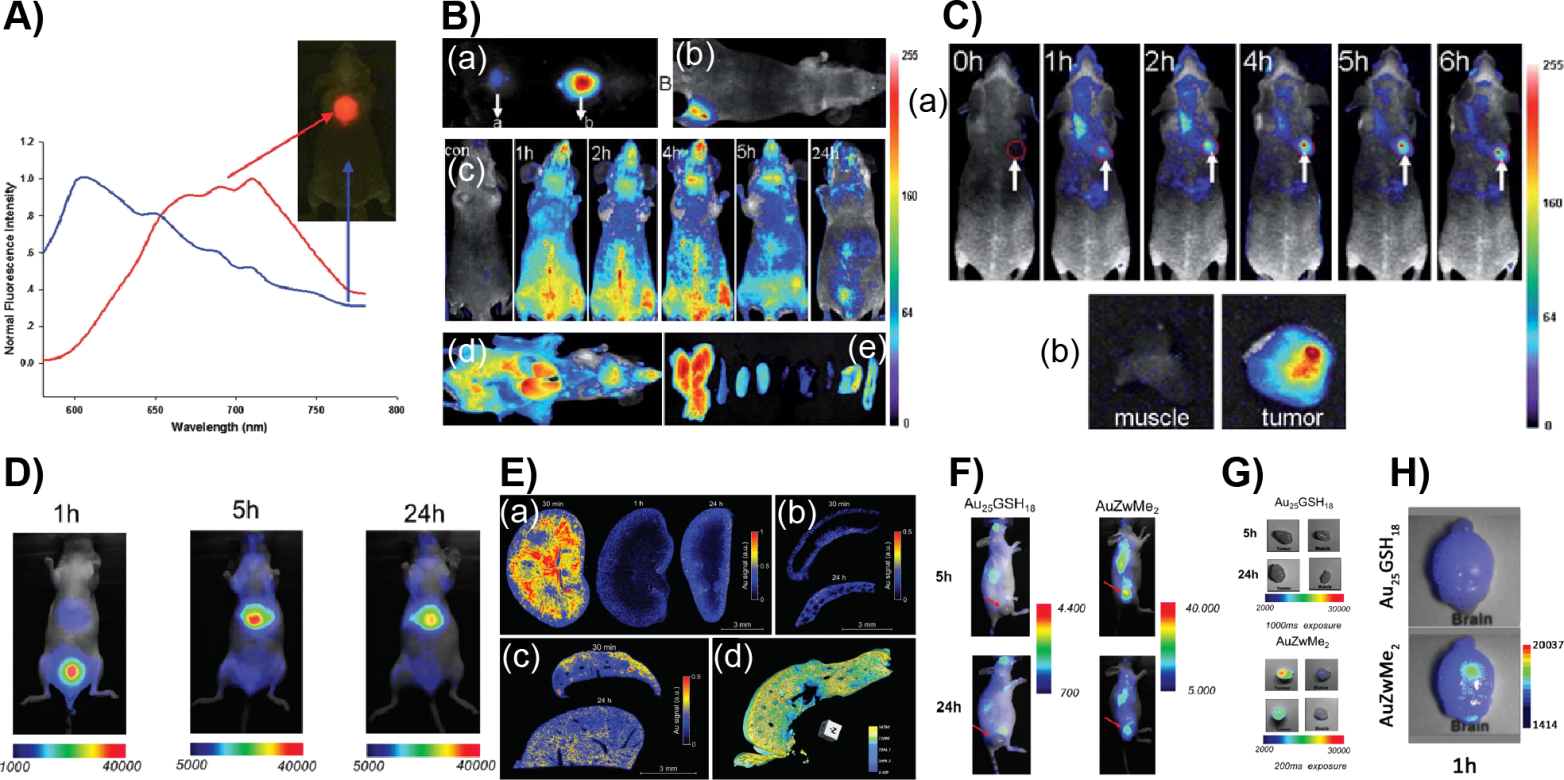
In vivo bioimaging using luminescent AuNCs. A) NIR fluorescence imaging of BALB/c mouse treated with the Au-BSA NCs. The blue line shows the autofluorescence and Au-BSA NCs signal in red in the fluorescence spectra. B) In vivo fluorescence image of Au-BSA NCs injected (a) subcutaneously and (b) intramuscularly into the mice, (c) post-injection real-time in vivo abdomen imaging of intravenously injected with AuNCs as a function of time, (d) ex vivo imaging of anatomized mice after injection of 200 mL of AuNCs and (e) some dissected organs (liver, spleen, left kidney, right kidney, heart, lung, muscle, skin, and intestine from left to right). C) (a) Fluorescence images of mice bearing an MDA-MB-45 tumor and (b) ex vivo fluorescence image of the tumor tissue and the muscle tissue around the tumor from the mice. D) In vivo whole-body fluorescence imaging at time intervals of 1, 5 and 24 h after intravenous injection of AuZwMe_2_. (E) (a) LIBS measurement of kidney slices 30 min, 1 h, and 24 h post-injection of AuZwMe_2_ NCs. LIBS measurement of (b) spleen and (c) liver slices 30 min and 24 h post-injection of AuZwMe_2_. (d) False-color 3D reconstruction of 600 μm thickness of a mouse liver 5 h post-injection of AuZwMe_2_ by means of X-ray phase-contrast tomography imaging. F) In vivo whole-body fluorescence imaging 5 and 24 h after intravenous injection of AuZwMe_2_ or Au_25_GSH_18_. G) Ex vivo fluorescence imaging of the tumor (top) and muscle (bottom) 5 and 24 h after AuZwMe_2_ and Au_25_GSH_18_ intravenous injection. H) Ex vivo fluorescence imaging of Au_25_GSH_18_ (top) and AuZwMe_2_ (bottom) in isolated orthotopic U87MG glioblastoma-bearing brains, 1 h post-injection. Figure panel 7A is reused and panels 7B,C are adapted with permission from [98], copyright 2010 The Royal Society of Chemistry. Figure panels 7D and 7F–H are reused, and panel 7E is adapted with permission from [99], copyright 2018 The Royal Society of Chemistry.

Conpared to cell-line and isolated in vitro studies, in vivo imaging using animal models faces additional challenges. This is attributed to an increased complexity, a decreased transmission of visible light through biological tissues, the interaction with various biomolecules and a possible degradation of the luminescent materials. However, NIR emission, biocompatibility, and photothermal stability make luminescent AuNCs potential candidates for in vivo imaging. Using NIR-emitting Au-BSA NCs, Wu et al. successfully demonstrated in vivo imaging in a mouse model [98]. The Au-BSA NCs were subcutaneously injected to test the efficiency of a localized signal under a few millimeters of tissue (Figure 7A,B). It was shown that a strong emission at 710 nm was easily separated from autofluorescence. The detection limit was found to be 0.235 mg/mL of AuNCs. Intravenous injection of Au-BSA NC solution to BALB/c nude mice allowed for real-time imaging of the whole body. The fluorescence emission of NCs was visualized in the superficial vasculature of the whole body immediately upon tail vein injection of NC solution. However, as the blood circulation continued, the emission intensity decreased gradually and remained visible up to 5 h post-injection. A notable decrease in luminescence was observed after 24 h in the whole body except liver and bladder, suggesting the clearance of the Au-BSA NCs through the urinary clearance system. Under ex vivo imaging conditions, the harvested organs, including liver, spleen, kidney, heart, lung muscle, skin, and intestine, showed a fluorescence comparable to that of the in vivo imaging at 5 h post-injection.

Guevel et al. reported AuNCs stabilized by zwitterionic molecules for subcutaneous and orthotropic glioblastoma mice models [99]. Two types of Au_25_ NCs were used, namely, glutathione-capped [Au_25_(SG)_18_] and lipoic acid-sulfobetaine zwitterion-capped [Au_25_(ZWMe_2_)_18_] NCs. Intravenous injection of [Au_25_(ZWMe_2_)_18_] and in vivo fluorescence imaging after 1 h showed a strong signal in the bladder indicating a high and fast renal clearance. Further, a strong fluorescence in the NIR region (that of NCs) was observed in urine generated during the first hour after injection (Figure 7D). This is an indication that the NCs are not metabolized in vivo and retain their structure upon excretion. Similar to many other studies, the fluorescence signal was still observed in the liver after 5 h and to a lesser extent after 24 h, presumably due to NC aggregation. The ex vivo imaging of organs harvested at 1, 5 and 24 h exhibited a low level of fluorescence in the kidney further showing renal clearance (Figure 7E). A decrease of 66% in the NC signal between 1 and 5 h was observed. Using multi-elemental laser-induced breakdown spectroscopy (LIBS) particle clearance and Au content in tissues were studied. A strong signal of Au was observed in the kidney within 30 min mostly in the medulla and decreased after 1 h with a weak residual cortical uptake. However, in the liver, a weak Au signal remained intact up to 24 h possibly due to the accumulation and internalization of NCs in Kupffer cells. Histological studies of the organs revealed that there are no necrotic cells or atrophic tubes or specific immunogenic infiltration between 5 and 24 h post-injection. This indicates that there is no acute toxicity. X-ray tomography confirmed the uniform distribution of Au in the liver. The tumor uptake studies were performed for [Au_25_(SG)_18_] and [Au_25_(ZWMe_2_)_18_] intravenously injected in mice bearing a subcutaneous U87MG tumor by tracking the NCs using fluorescence imaging. No signal was detected after 5 or 24 h for [Au_25_(SG)_18_]. However, strong fluorescence was observed for [Au_25_(ZWMe_2_)_18_] in the tumor after 5 h and a slight decrease after 24 h. The tumor-to-skin ratio was determined after 1 and 24 h. It was found to be higher for [Au_25_(ZWMe_2_)_18_] and remained constant. To further validate the uptake in orthotropic brain tumors, NCs were injected into mice bearing U87MG tumors engrafted in the brain (Figure 7F). Again, [Au_25_(ZWMe_2_)_18_] was found to yield a three times stronger signal than [Au_25_(SG)_18_] at 1 h post-injection. Chen et al. have shown that zwitterionic LA-sulfobetaine-capped AuNCs can be used for in vivo shortwave infrared imaging using a mouse model [100]. Li et al. reported nanoparticle assemblies of pea protein isolate (PPI)-capped AuNCs with red fluorescence for in vitro and in vivo imaging. The nanoparticles were coated with red blood cell membranes to improve their blood circulation and enhance their enrichment in tumors [101].

**Figure 8:**
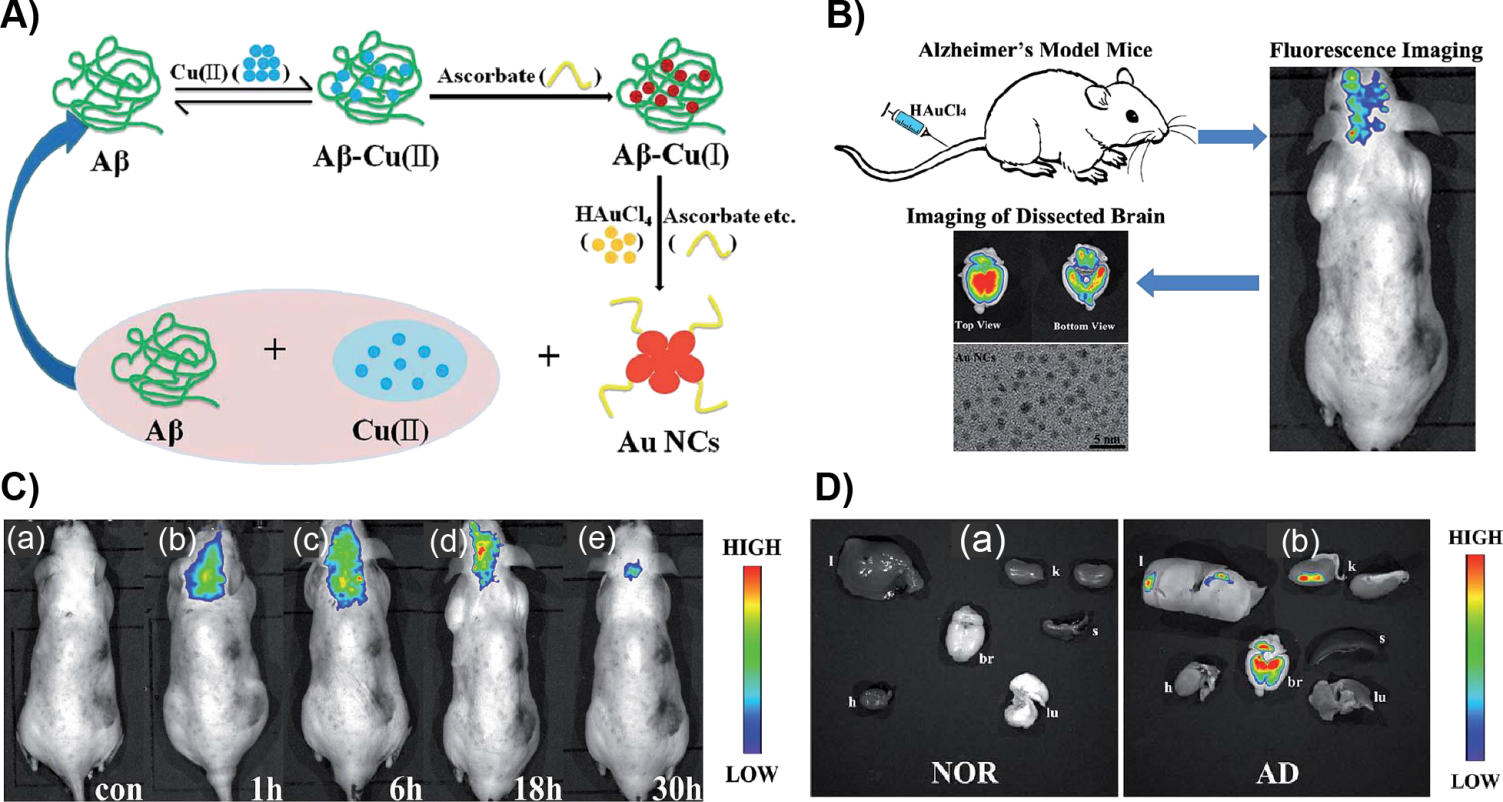
A) Principle of Aβ/copper and ascorbate- or Fe(II)-catalyzed formation of AuNCs. B) Illustration of fluorescence biomarking of mice brain with Alzheimer’s disease. C) Fluorescence imaging of (a) the control group of AD mice (without given HAuCl_4_). (b–e) Fluorescence imaging of the same AD model mice after tail-vein injection of HAuCl_4_ solution after different periods of time (1, 6, 18 and 30 h). D) Fluorescence imaging of harvested organs (l: liver, h: heart, br: brain, k: kidney, s: spleen, and lu: lung) of the control group and of AD model mice after tail-vein injection of HAuCl_4_ solution at 30 h post-injection. Figure panels 8A and 8B are reused and panels 8C and 8D are adapted with permission from [102], copyright 2016 The Royal Society of Chemistry.

Lai et al. reported the in vivo formation of fluorescent gold nanoclusters for imaging the brain affected by Alzheimer’s disease (AD) [102]. The redox microenvironment in the AD brain is characterized by relatively low oxygen metabolism and more free radicals compared to that of a healthy brain. When AD occurs, a large amount of ascorbate and an elevated level of hydrogen peroxide, other free radicals and redox ligands appear at the lesions in the brain. Thus, HAuCl_4_ ions accumulated in the hippocampus can be can be potentially reduced. After tail-vein injection into four-month-old APP/PS1 male mice, imaging was performed in vivo. For ex vivo imaging, the harvested organs of mice at 30 h post-injection were used. The maximum fluorescence appeared 18 h after injection. Afterward, the fluorescence signal and imaging area decreased. However, the control group did not show any fluorescence further allowing for selective formation and imaging (Figure 8).

## Conclusion

The unique chemical, optical and catalytic properties of gold nanoclusters have led to rapid progress in their application. This can be attributed to tunable photoluminescence, low toxicity, high bioavailability and renal clearance. The studies on bioimaging based on gold nanoclusters started only a decade ago. However, progress has already been made regarding potential applications in the rapid sensing of biomolecules and pathogens, in vitro imaging of various cell lines, and in vivo imaging. Appropriate modifications also offer an opportunity for the rational design of nanocarriers encapsulating poorly soluble drugs for targeted delivery. Cell line-based studies have provided enough evidence that the internalization of AuNCs occurs through endocytosis. However, there are several challenges to utilize AuNCs in bacterial sensing due to their inability to distinguish different strains. Similarly, AuNC-based radiotherapeutic applications are not able to distinguish healthy mammalian cells from infected cells. Further, in vivo imaging has provided crucial insights on uptake and excretion, which are comparably fast. In a majority of the cases, Au_25_NCs have been utilized. Furthermore, mostly protein-coated or glutathione-capped NCs have been studied. It is important to note that currently only a limited number of AuNCs with an acceptable level of PL and quantum yield are reported. Like many other nanomaterials, AuNCs have shown not to undergo metabolism inside the body. Hence, the accumulation in the liver is still one of the concerns. The fate of accumulated AuNCs in the liver is unknown. Corresponding studies will be useful to realize the development of new bioimaging methods and their practical applications.
